# On the limits of graph neural networks for the early diagnosis of Alzheimer’s disease

**DOI:** 10.1038/s41598-022-21491-y

**Published:** 2022-10-21

**Authors:** Laura Hernández-Lorenzo, Markus Hoffmann, Evelyn Scheibling, Markus List, Jordi A. Matías-Guiu, Jose L. Ayala

**Affiliations:** 1 Department of Computer Architecture and Automation, Computer Science Faculty, Complutense University of Madrid, 28040 Madrid, Spain; 2 Department of Neurology, Hospital Clínico San Carlos, San Carlos Research Health Institute (IdISSC), Universidad Complutense, 28040 Madrid, Spain; 3 Big Data in BioMedicine Group, Chair of Experimental Bioinformatics, TUM School of Life Sciences, Technical University of Munich, Munich, Germany; 4 Institute for Advanced Study, Technical University of Munich, Lichtenbergstrasse 2 a, 85748 Garching, Germany

**Keywords:** Machine learning, Predictive medicine

## Abstract

Alzheimer's disease (AD) is a neurodegenerative disease whose molecular mechanisms are activated several years before cognitive symptoms appear. Genotype-based prediction of the phenotype is thus a key challenge for the early diagnosis of AD. Machine learning techniques that have been proposed to address this challenge do not consider known biological interactions between the genes used as input features, thus neglecting important information about the disease mechanisms at play. To mitigate this, we first extracted AD subnetworks from several protein–protein interaction (PPI) databases and labeled these with genotype information (number of missense variants) to make them patient-specific. Next, we trained Graph Neural Networks (GNNs) on the patient-specific networks for phenotype prediction. We tested different PPI databases and compared the performance of the GNN models to baseline models using classical machine learning techniques, as well as randomized networks and input datasets. The overall results showed that GNNs could not outperform a baseline predictor only using the APOE gene, suggesting that missense variants are not sufficient to explain disease risk beyond the APOE status. Nevertheless, our results show that GNNs outperformed other machine learning techniques and that protein–protein interactions lead to superior results compared to randomized networks. These findings highlight that gene interactions are a valuable source of information in predicting disease status.

## Introduction

Neurodegenerative diseases (NDDs) are complex diseases showing progressive neuronal degeneration and death in the central nervous systems. The most common cause of NDDs is Alzheimer’s Disease (AD), a progressive brain disease that mainly affects cognitive functions and usually starts with memory issues^1^. Its pathological features are the accumulation of amyloid-β plaques and tau neurofibrillary tangles. The etiology of AD is unclear, but several risk factors are considered: aging, genetics, family history, and modifiable risk factors such as education years, physical activity, or smoking^1^. The apolipoprotein E (APOE) gene is the major risk factor for AD, with various alleles encoding for different isoforms of the protein. APOE ε4 is the most risk-related isoform, significantly associated with late-onset AD (LOAD)^2^. However, this isoform has also been found in non-dementia subjects, suggesting that other genetic factors trigger the disease.

Precision medicine is a new approach in medicine whose main objective is to personalize the diagnosis and treatment of a patient. In particular, genomics holds the potential to identify risk factors that can be used for early diagnosis, which is crucial for NDDs, where the underlying biological mechanisms are triggered long before the onset of symptoms^3^. Early diagnosis of NDDs can justify regular neuroimaging follow-ups and possible treatments to prevent or delay the onset of the disease.

Typically, the study of the genetic basis of diseases has followed a strategy focused on selecting one or several genes to find genetic variants that are established, with greater or lesser penetrance, as the genetic cause of the disease^4^. This strategy has significantly eased the understanding of the genetic architecture of many diseases. However, when studying polygenic diseases such as AD, the genetic complexity is hard to explain using one-gene approaches. Therefore, handling information about several affected genes, their interactions, and related products seems necessary. In this regard, approaches related to systems biology, specifically network-based analyses, are crucially needed to study the genetic architecture of complex diseases^5^. These approaches propose genetic variants as perturbations that can modify the topology of the studied network^6^. Moreover, since the prediction of a phenotype through genetic information can be considered a high dimensional problem—that is known to be problematic to identify patterns—we present strategies based on machine learning (ML) algorithms as a solution to phenotype prediction.

The development of ML techniques and the availability of genetic cohorts during the last decade has created interest towards ML-based phenotype prediction. In particular, deep learning (DL) models appear suited for this complex prediction task since they can model the nonlinear interactions of several genetic factors that rise to complex diseases^7^. For instance, DeepBipolar^8^ uses Convolutional Neural Networks (CNNs) to predict bipolar disorder from exome variants. Since the main challenge in creating robust prediction models is the high dimensionality and co-linearity of genomics datasets, several works have focused on feature selection or extraction techniques as a pre-processing step. To reduce the number of features, we can leverage prior knowledge and select only those genetic variants or genes for analysis that have previously been associated with the disease^9,10^. However, these approaches discard the biological interactions or relationships between these genes or proteins, which may contribute to the disease mechanisms. We propose that prior information about such interactions, captured in biological networks such as protein–protein interaction (PPI) networks, co-expression networks, or metabolic pathways, should be used for building phenotype prediction methods for NDDs.

Since graph-structured data is prevalent across different disciplines, DL strategies that can handle such data have been developed in recent years. Graph Neural Networks (GNNs) are a relatively new type of neural networks that operate on graph-structured data and have successfully been used in network biology applications, such as proteomics, drug development or discovery, polypharmacy, metabolic pathways analysis, or disease diagnosis^11^.

This study presents a genotype-to-phenotype prediction pipeline that uses GNNs in combination with biological networks (PPIs and functional networks) related to AD. We employed this pipeline in different AD-cohorts: the Alzheimer’s Disease Neuroimaging Initiative (ADNI) genetic cohort of 808 subjects and the TGenII GWAS dataset with 1599 subjects. To the best of our knowledge, this is the first work integrating information from genetic variants into a biological network for disease prediction through Graph Neural Networks, applied in the context of neurodegenerative diseases. We aim: (i) to evaluate if GNNs are suitable ML models for phenotype prediction using genotype data, (ii) to assess the influence of using different biological networks for building the input graph datasets for GNNs, (iii) to test whether this phenotype prediction could benefit from structuring the information in a known biological network, and (iv) to assess limitations and opportunities for predicting AD-related phenotypes.

## Materials and methods

**Figure 1:**
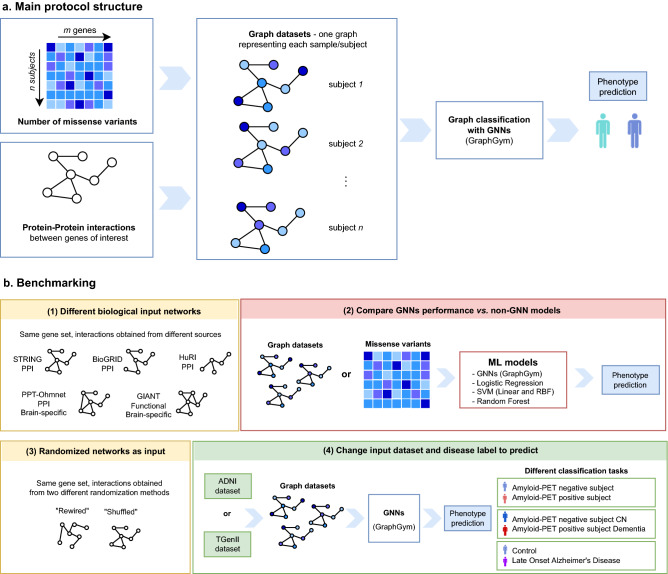
(**a**) Overview of the workflow for phenotype prediction through genotype data. Starting with a set of genes of interest to study, we extracted the number of missense variants in each gene in a genetic cohort and built a protein–protein involving these genes. With the input networks and genetic data, we built different graph datasets, in which each graph represented a sample, including the number of missense variants as node attributes. These graph datasets are lastly used as input for phenotype prediction using Graph Neural Networks (GNNs), evaluated on a framework called GraphGym^12^. (**b**) Overview of the benchmarking. We compared the performance of GNNs (1) using graph datasets coming from different biological networks, (2) against other non-GNN models using the number of missense variants, (3) using graph datasets coming from randomized networks, and (4) for predicting of several labels from different input datasets.

In this work, we present a pipeline for predicting AD-related phenotypes using biological networks and genotype information through GNNs. We further performed extensive benchmarking work on how GNNs work with different input networks, with respect to other ML models, or using different datasets in the context of AD prediction. Figure 1 summarizes the methods employed in this work. First we show the main procedure for building graph datasets in Fig. 1a. Secondly, Fig. 1b summarizes the extensive benchmarking of GNNs in AD-related phenotypes prediction. We compared five different PPI databases for extracting disease-related subnetworks as input for GNNs and compared their performance against classical machine learning methods that leverage only the genotype information of the nodes, disregarding their interactions. The tested models included Logistic Regression, Support Vector Machine (SVM) with Linear and Radial Basis Function (RBF) kernels, and Random Forest. Finally, we evaluated the importance of the network structure and topology in GNNs models' performance, by evaluating graph datasets built on random networks. We performed these steps using the ADNI cohort and two different labelings for classification: (i) amyloid positron emission tomography (PET) status (positive or negative), and (ii) amyloid-PET status together with the diagnosis (AD with positive amyloid-PET vs. Cognitively Normal with negative amyloid-PET). Lastly, we applied these steps to the TGenII cohort to see if we could expect similar classification behavior of GNNs using a completely different but also AD-related dataset. The manuscript is organized as follows. In Section “Materials and methods”, we describe the steps that form the proposed phenotype prediction pipeline (Fig. 1a) and the benchmarking steps carried out to evaluate it (Fig. 1b). In Section “Results”, we report the outcomes of the main pipeline and benchmarking. Finally, in Section “Discussion”, we discuss the results obtained mainly from the benchmarking point of view, going into their implications in the context of Alzheimer's Disease prediction.

### Data generation and filtering

For this work, we made a prior selection of disease-associated genes that were extracted from DisGeNET^13^, a large database that collects associations of genes and variants to diseases. We obtained 101 genes associated with Alzheimer’s Disease (AD). Among all gene-disease associations obtained, we considered only those labeled in DisGeNET^13^ as coming from “curated” source databases.

We employed two independent genetic datasets to test this protocol: (i) the genetic cohort from the Alzheimer’s Disease Neuroimaging Initiative (ADNI) and (ii) the TGenII GWAS dataset from NIAGDS. The ADNI (adni.loni.usc.edu) was launched in 2003 as a public–private partnership led by Principal Investigator Michael W. Weiner, MD. The primary goal of ADNI is to test whether serial magnetic resonance imaging, PET, other biological markers, and clinical and neuropsychological assessment can be combined to assess the progression of Mild Cognitive Impairment and early AD. The ADNI genetic cohort contains 808 subjects, whereas the TGenII cohort contains 1599. In both datasets, we first extracted genetic variants mapping to any of the genes of interest using GRCh37 genetic coordinates. Using Ensembl’s Variant Effect Predictor (VEP)^14^, we functionally annotated these variants. Lastly, using the “split-vep” plugin from BCFTools^15^, we selected from the annotated VCF files only missense variants.

We labeled each dataset differently for later use as prediction tasks in classification (Fig. 1b). Regarding ADNI dataset, the first prediction task, named “PET”, represented each subject's last visit amyloid-PET status. Using the “ADNIMERGE” table from ADNI, we queried each subject’s last visit Florbetapir or Pittsburgh Compound-B (PiB) standardized uptake value ratio (SUVR). Then we applied threshold values of 1.11 and 1.27^16–18^ for Florbetapir or PiB SUVR measurements, respectively, to obtain the positive or negative amyloid PET status of each subject’s last visit. The second prediction task, named “PET&DX” represented the last visit amyloid-PET status together with the diagnosis of each subject. This labeling corresponded to a subset of the first-mentioned one since it also considered the clinical diagnosis of each individual. PET&DX labeling included two types of subjects: amyloid PET positive individuals with a Dementia diagnosis, and amyloid PET negative individuals without cognitive impairment, identified as Cognitively Normal in ADNI. We decided to employ amyloid-PET status because of the heterogeneous nature of AD diagnosis. AD diagnosis could include controls probably misclassified as AD and non-AD dementias misclassified as probable AD^19^. Moreover, cognitive symptoms of AD are developed much later than the amyloid-PET status, which can be detected up to a decade before the development of this cognitive impairment. Lastly, regarding the TGenII dataset, we used the case or control labeling of the original data set with labels for Late-Onset Alzheimer’s Disease case (LOAD) or control.

### Generation of networks

#### Biological networks

Once the genes of interest were selected (see Section “Data generation and filtering”), we obtained molecular interactions between them. To this end, we queried multiple databases, allowing us to assess the importance of the network employed. In addition to the well-known interaction databases STRING^20^ and BioGRID^21^, we also queried The Human Reference Interactome (HuRI)^22^, PPT-Ohmnet^23^, and GIANT^24^. From STRING^20^ (v.11.5), we selected protein–protein physical interactions from the source databases, text mining, and predicted using the default confidence score. From BioGRID^21^ (v.4.4.206), we selected protein–protein human interactions from high-throughput experiments. HuRI^22^ (downloading date: March 2022) is a recent database storing only high-quality binary interactions between proteins, coming from a systematic experimental screening pipeline that included at least two types of experimental evidence. The PPT-Ohmnet^23^ (downloading date: March 2022) network stores protein–protein physical interactions specific to different human tissues based on co-expression information. We queried for brain-specific interactions between our genes of interest. Lastly, GIANT^24^ (downloading date: March 2022) is a tissue-specific predicted functional network. We selected only interactions found in brain tissue using the "gold standard" brain file restricted to functional edges between genes specifically coexpressed in the brain from GIANT. In all the described cases, we queried only for AD-related genes extracted from DisGeNET and kept only the largest connected component. Regarding this restriction, we lost a percentage of the original number of genes of interest, which varied between the different networks. From highest to lowest loss (number of lost nodes/original number of nodes, percentage): PPT-Ohmnet (29/45, 35.56%), HuRI (6/18, 33.33%), GIANT (9/45, 20%), BioGRID (8/48, 17.39%), and STRING (7/59, 11.86%). Among all the sources described, the most curated or high-quality interaction information is found in HuRI. Lastly, there are only two sources with brain-specific interactions: PPIs from PPT-Ohmnet and functional interactions from GIANT.

The different subnetworks extracted as described above serve as "templates" for generating graph datasets, i.e. a network defining interactions between genes which we can further fill with different nodes (genes) attributes. Table 1 summarizes the graph properties of all the biological networks used as templates. For the remaining manuscript, we use the following naming convention for the biological networks to differentiate them from the interaction databases used: the first part describes the gene set and the second part describes the interaction database from which it comes, e.g. “AD STRING” refers to AD-related genes interacting in a PPI network coming from STRING.

**Table 1:** Properties of the biological networks.

Network name	Description	Network description
AD STRING	Genes related with Alzheimer’s Disease PPI network	52 nodes; 111 edges density: 0.0837; diameter: 6
AD BioGRID	Genes related with Alzheimer’s Disease PPI network	38 nodes, 57 edges density: 0.0810; diameter: 5
AD HuRI	Genes related with Alzheimer’s Disease PPI network	12 nodes, 13 edges density: 0.1969; diameter: 6
AD PPT-Ohmnet	Genes related with Alzheimer’s Disease PPI network, brain-specific	29 nodes, 52 edges density: 0.1281; diameter: 6
AD GIANT	Genes related with Alzheimer’s Disease Functional network, brain-specific	36 nodes, 100 edges density: 0.1587; diameter: 6

#### Random networks

As shown by Lazareva et al.^25^, the performance that obtains randomly perturbed networks in disease module mining often show comparable performance to the original networks. To make sure that the selected interactions offer additional value to the selected genes, we thus built random networks for the network that we selected as the “best”, meaning the network whose graph dataset obtained a better result for phenotype prediction. We used two different randomization algorithms named "Shuffled" and "Rewired" implemented by Lazareva et al.^25^. Randomization algorithms are methods that randomly alter the topology or elements (nodes or edges) of a network. The two randomization algorithms mentioned differ in how they randomly alter the original network. The "Shuffled" algorithm shuffles the node labels while maintaining the original network topology. The "Rewired" algorithm rewires the edges maintaining the original degree of each node. For further information, see Lazareva et al.^25^. For each randomization method, we generated 100 different random networks and built for each of them their corresponding graph dataset.

### Creating patient-specific graphs from genetic profiles

We used the networks described in Section “Generation of networks” as "templates" (both biological and random networks) for generating graph datasets. We built these datasets of patient- or subject-specific graphs for each cohort using the genetic and phenotypic data for supervised classification in graph neural networks. In each subject’s graph, we annotated the number of missense genetic variants per gene (see Section “Data generation and filtering”) as node attributes. We labeled each subject’s graph with the different labels described in Section “Data generation and filtering” for each of the cohorts. Accordingly, as all the labels were binary, we assigned the value 1 for the positive class (case/disease) and 0 for the negative class (control/healthy). Considering the five different biological networks used and the two different labels of the ADNI cohort, we generated a total of ten different graph datasets that we initially evaluated during this work (see Section “Comparing results using different input networks”). To this total number should also be added the 200 (2 × 100) graph datasets created for the random networks (see Section “Using randomized networks as input”) and the graph dataset created for the LOAD label of the TGen II cohort (see Section “Using another dataset as input”).

### Supervised classification

#### Train and validation sets used

We split the generated datasets into ten different training and test sets by means of a stratified tenfold split. The stratified split makes sure the proportion of labels in all the folds is the same or very similar to the proportion of labels in the complete dataset. All the models evaluated throughout this manuscript were trained and tested with these ten splits.

#### Graph classification with graph neural networks

GNNs are a type of deep neural network architecture that can operate over graph-structured data^26^. GNNs mainly work to obtain a new feature space, also known as node embeddings, representing both nodes and any other information found in the graph.

We trained and evaluated GNNs in the framework GraphGym^12^, tuning hyperparameters as the configurations in the design space as recommended by its authors^12^ (see Supplementary Table 1), with two exceptions: considering the small diameter of the PPI networks employed here, we set the number of message passing layers to two and the number of epochs to 200 to avoid over-smoothing. Each experiment, meaning applying a GNN model or configuration to a dataset or task, is repeated three times (using three different random seeds). At the end of each experiment, GraphGym produces the mean and standard deviation of several performance metrics obtained in the three repeats for the training and validation sets. We ran GraphGym for each of the 10 different splits (see Section “Train and validation sets used”) and obtained the best GNN configuration in each split. Then we obtained the best GNN configuration across the ten folds and reported their results in the corresponding test sets over the ten different folds in terms of mean accuracy, precision, recall, F1-score, and area under the ROC curve (AUC). See Supplementary Information Sect. 1.1 for more details about GraphGym use.

#### Canonical machine learning models

To compare the GNN results with other models that did not input graph-structured data, we applied Logistic Regression, SVMs with Linear and RBF kernels, and Random Forest to our data. It is important to note that in this case, the data was structured in a tabular way, with no interaction information between the genes. The idea behind this benchmark was to assess whether including interactions data, by using GNNs, was an advantage in phenotype prediction. For each of the mentioned algorithms, we performed nested cross-validation. For each of the described folds in Section “Train and validation sets used”, we obtained the best models’ hyperparameters by means of a 10-Fold cross-validation grid search on the training samples of that fold. The grid of hyperparameters evaluated for each of the algorithms are listed in Supplementary Table 2. We then evaluated this best model using the test set from that fold. Therefore, we performed 100 (10 × 10) runs for each of the machine learning algorithms described. As done with GNNs, we report the mean and standard deviation of accuracy, precision, recall, F1-score, and AUC on the test set over the ten different folds.

### APOE gene significance assessment

Due to the high importance of the APOE gene in AD or related endophenotypes we tested its influence on the labels to be predicted. Firstly we built a Logistic Regression model that took only the APOE gene as input. We refer to these models as “baseline”, as they show what we can expect from the datasets by including a single gene closely related to AD. Secondly, because we expected this gene to greatly influence the classification regarding its high correlation with AD-related phenotypes, we also evaluated all models' performance using datasets with and without this gene. We decided to delete APOE from the datasets to test whether there could be other genes influencing the development of the disease. This fact is especially important to unravel why some patients develop AD without having mutations in the APOE gene. Conversely, subjects without AD with the APOE gene affected might have variants in other genes that protect against the development of the disease.

### Implementation

All the steps previously described are implemented in Python 3 and R 4.1. The scripts used in each step are available at https://github.com/laurahdezlorenzo/graph_genetics under a MIT License. Additional specifications about GNNs and methods described in this manuscript are described in an AIMe report: https://aime.report/thfKRo. Gene-disease associations were obtained using disgenet2r^13^. Genomic coordinates of the genes were obtained using biomaRt^27^. Variants from the VCF files were extracted using genomic coordinates employing VCFTools v.0.1.16^28^ and functionally annotated using Ensemble’s VEP v.105.0^14^. Missense variants obtained in the genes of interest were extracted using BCFTools’ v.1.11^15^ plugin “split-vep”. Graph datasets were built using Python’s NetworkX v.2.6.3 library^29^. GNNs were tested using GraphGym v.0.2.0^12^. Machine learning models were implemented using Scikit-Learn v.1.0.2 library^30^. Lastly, statistical analyses were done using SciPy v.1.7.3^31^.

## Results

The results obtained are organized as follows. Section “Comparing results using different input networks” describes the results obtained with GNNs using different graph datasets obtained from the different biological networks described in Section “Biological networks” to select the best network for building graph datasets among the five candidates. Section “Benchmarking GNNs performance vs. canonical machine learning models” compares the results obtained with the best-performing graph dataset against other non-GNN models. In Section “Using randomized networks as input”. we evaluated the GNNs classification performance on graph datasets coming from random networks to assess the importance of real interactions and the overall topology of the biological networks in classification. We performed these sections using the ADNI dataset with two different classification labels: PET and PET&DX. Lastly, in Section “Using another dataset as input”, we applied some of these previous steps using the TGenII dataset for LOAD prediction. This last section aimed to corroborate if we could expect similar results from an independent dataset and observe the differences in prediction using different classification labels.

### Comparing results using different input networks

**Figure 2:**
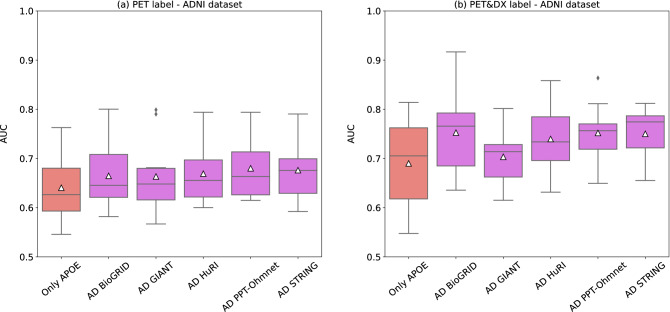
Boxplots showing the AUC distribution on the validation set for different GNN models using different graph datasets built on different networks using as targets: (**a**) PET, or (**b**) PET&DX. Red boxplots (labeled as “Only APOE”) correspond to the baseline models. White triangles (△) represent mean AUC values.

Using the five different input interaction networks built (AD BioGRID, AD GIANT, AD HuRI, AD PPT-Ohmnet, and AD STRING), and two different labels from ADNI cohort (PET and PET&DX) led to ten different classification tasks that we evaluated using GNNs. Supplementary Table 3 shows the best GNN configurations and their mean performance values in the validation set for each graph dataset and classification task proposed. Figure 2 shows the distribution of AUC values on the validation set over the ten splits.

Regarding the PET label (Fig. 2a, Supplementary Table 3a), results showed that the mean AUC performance was slightly higher for all networks (AD BioGRID: 0.6648 ± 0.0656, AD GIANT: 0.6635 ± 0.0786, AD HuRI: 0.6692 ± 0.0624, AD PPT-Ohmnet: 0.6801 ± 0.0643, AD STRING: 0.6763 ± 0.0637) compared with the baseline model (0.6406 ± 0.0691). Among all the graph datasets tested, the one that obtained the best AUC value was the one built using the brain-specific PPI: AD PPT-Ohmnet. However, when performing the 1-sample t-tests against the PET baseline model result, the mean AUC values of all graph datasets were not significantly better than the baseline model. Although the p-values obtained were not significant, we used them as a ranking score of the datasets to select the best graph dataset. Therefore, in the case of the PET label, the ranking for each graph dataset resulted as follows: AD PPT-Ohmnet (0.1007), AD STRING (0.1224), AD HuRI (0.1718), AD BioGRID (0.21595), AD GIANT (0.2485).

Regarding the PET&DX label (Fig. 2b, Supplementary Table 3b), the mean AUC values of all graph datasets (AD BioGRID: 0.7526 ± 0.0819, AD GIANT: 0.7035 ± 0.0634, AD HuRI: 0.7397 ± 0.0682, AD PPT-Ohmnet 0.7521 ± 0.0589, and AD STRING: 0.7502 ± 0.0563) were better than the baseline model (0.6901 ± 0.0979). The graph dataset that performed best was again AD PPT-Ohmnet. In the case of this graph dataset, the p-value obtained was very near to significance (0.05171). The p-values ranking for this label was: AD PPT-Ohmnet (0.0517), AD STRING (0.0547), AD BioGRID (0.0692), AD HuRI (0.1025), and AD GIANT (0.3604).

The PET&DX prediction task led to better results than the PET labeling. In both cases, AD PPT-Ohmnet and AD STRING were ranked first and second, and the GIANT brain functional network was always ranked the last. Therefore, for both labels, we selected the AD PPT-Ohmnet network for further analysis.

### Benchmarking GNNs performance *vs*. canonical machine learning models

**Figure 3:**
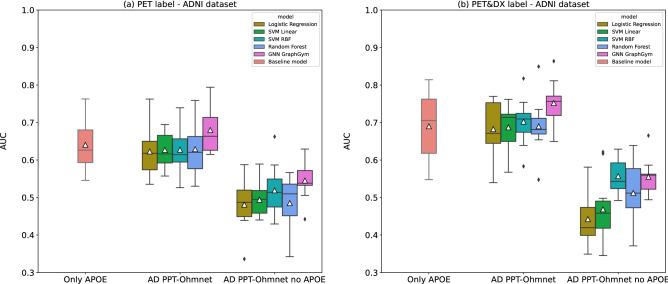
Boxplots showing the AUC distribution values of different models on the test set using as targets either (**a**) PET, or (**b**) PET&DX. Red boxplots (labeled as “Only APOE”) correspond to the baseline models. White triangles (△) represent mean AUC values.

We then compared the performance of the best GNN models obtained in the previous section to Logistic Regression, SVMs with Linear and RBF kernels, and Random Forest. Supplementary Table 4 shows the performances of all these models in terms of accuracy, precision, recall, F1-score, and AUC. Note that the AD PPT-Ohmnet network structure did not vary much when deleting the APOE gene (28 nodes, 46 edges, density 0.1217, diameter 6). Therefore we used the same best GNN configuration found for each label (PET and PET&DX) for the AD PPT-Ohmnet graph dataset (see Supplementary Table 3). Figure 3 shows AUC values distributions obtained on the validation set over the ten splits in each label used in the ADNI dataset. According to these performance results, we performed several statistical tests. Firstly, we tested whether these canonical machine learning models’ performances were significantly better than (i) the corresponding baseline model (t-test) and (ii) a random prediction (AUC value of 0.5, 1 sample t-test). Secondly, we tested if non-GNN models were significantly worse than the corresponding GNN model (t-test). Lastly, we compared if models without the APOE gene were significantly worse than those with it (t-test). Supplementary Table 5 summarizes all the obtained p-values.

When focusing on the PET label (Fig. 3a), the models that obtained a better mean AUC value were the GNNs, both in the case of datasets with APOE and those without. All non-GNN models performed significantly worse than their corresponding GNN model, except for Random Forest with the APOE gene and SVM RBF with the APOE gene (see Supplementary Table 5). However, neither GNNs nor non-GNN models significantly outperformed the corresponding baseline model. Furthermore, it is interesting to see that in the case of all models, when we removed the APOE gene from the input, their AUC performance significantly dropped compared to models with APOE. None of the models in the case of PET got significantly worse values than a random AUC of 0.5 (see Supplementary Table 5).

In the case of the PET&DX label (Fig. 3b), all models obtained a higher performance than the PET label but showed similar behavior to this label, with some exceptions. Again, all non-GNN models obtained a significantly worse AUC value than the corresponding GNN models, except for SVM RBF and Random Forest in the datasets without the APOE gene (see Supplementary Table 5). The models that obtained better performance in terms of mean AUC were the GNN in the dataset with APOE and SVM RBF and GNN models in the case of datasets without APOE. In the latter case, SVM RBF and GNN models obtained a very similar average performance (0.5571 ± 0.0481 and 0.5554 ± 0.0485, respectively, see Supplementary Table 4). Again, regarding datasets without the APOE gene, all the models obtained a significantly worse performance than models with APOE. Moreover, none of the models obtained a significantly worse value than random (AUC 0.5), except for Logistic Regression without APOE (see Supplementary Table 5). Lastly, as was the case in the PET label, none of the tested models performed significantly better than the baseline one. However, the GNN obtained a very near-significance p-value (5.1717e−02).

In all cases, GNN models showed better performance than non-GNN models. However, not all non-GNN models were significantly worse than the GNN ones. Importantly, the value obtained by the corresponding baseline models was not improved by any of the other models in any case. Furthermore, when we removed the APOE gene from the datasets, the performance of all models (both GNN and non-GNNN) worsened significantly, highlighting the importance of APOE as a biomarker for AD.

### Using randomized networks as input

Compared to other non-GNN models, GNNs were the models that obtained the best classification performances in the classification task proposed, although they did not obtain a significantly better performance than the baseline model using only the APOE mutation status. However, structuring the genetic data in a graph or network model such as the GNNs may still yield additional insights into the biology behind the disease, so we wanted to understand if the interactions present only in the graph datasets contribute to this.

**Figure 4:**
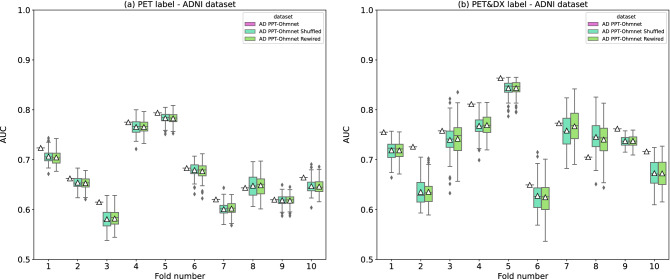
Boxplots showing the distribution of AUC values over the ten different test sets (ten different folds) using (i) the original AD PPT-Ohmnet dataset (pink), (ii) 100 different “shuffled” datasets (light blue), and (iii) 100 different “rewired” datasets (light green). White triangles (△) represent mean AUC values. Asterisks (*) represent a p-value < 0.05 (Benjamini–Hochberg correction) obtained by comparing original graph datasets’ performance *vs.* random graph datasets' performance. Boxplots were obtained for (**a**) PET and (**b**) PET&DX labelings using the ADNI dataset.

We selected the network AD PPT-Ohmnet as the best network candidate (see Section “Comparing results using different input networks”). To evaluate the importance of the interactions present in AD PPT-Ohmnet, we generated random networks from it. Then, we built their corresponding graph datasets and tested them in the proposed classification tasks with GNNs. These random networks involved the same sets of nodes (genes) of the original AD PPT-Ohmnet network but with different edges (interactions). We generated 100 different random networks for two randomization methods (“Shuffled” and “Rewired”, see Section “Random networks”) implemented by^25^, and built their corresponding graph datasets. These datasets were used for training and testing the original best GNN configurations obtained (Supplementary Table 3). We obtained a p-value for each fold, comparing the original AUC value in that fold against the 100 AUC values obtained using 100 different random graph datasets. We obtained these p-values through 1-sample one-sided t-tests, adjusted by the Benjamini–Hochberg method^32^. Figure 4 shows the distribution of random datasets' performance against the original datasets in each fold. Supplementary Table 6 includes all the p-values obtained here.

Nearly all the p-values obtained for the PET label were significant (p-value < 0.05), except for the case of the folds number 8 and 9. Regarding the PET&DX label, the results were practically the same: all the folds obtained a significant p-value except for fold number 8. The fact that the majority of the folds yielded a significant p-value suggests that the specific topology of the PPI networks played an important role in both labels prediction.

### Using another dataset as input

**Figure 5:**
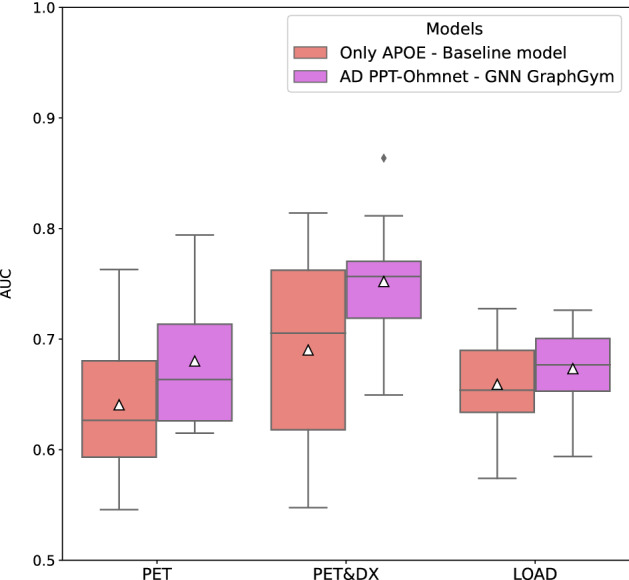
Boxplots showing the AUC distribution values of baseline and GNN models on the test sets using as targets either PET&DX, PET from the ADNI cohort, or LOAD from the TGenII cohort. White triangles represent mean AUC values.

Finally, we performed the steps described above using a completely different cohort: TGenII (see Section “Data generation and filtering”). This cohort contains more individuals (1599) than the ADNI cohort (808). In this case, the classification task was to predict the Late-Onset Alzheimer’s Disease (LOAD) diagnosis. We used the network AD PPT-Ohmnet to generate the corresponding graph dataset and obtained the best GNN model over the ten corresponding dataset splits (see Section “Train and validation sets used”) and its classification metrics on the validation set. As was the case in the previous scenarios with ADNI datasets, the GNN model was the one that obtained the best AUC results (0.6733 ± 0.0409, see Supplementary Table 8). The other models obtained AUC values very similar to the baseline model, with a value of 0.6591 ± 0.0451. However, as with the ADNI cohort, none of these models, including the GNN, obtained a significantly higher value (1-sample t-test, see Supplementary Table 9) than the baseline model, although all were significantly higher than a random AUC performance. We wanted to compare these results with the ones obtained in ADNI. Figure 5 shows the AUC distribution over the ten splits in the three different classification labels used (PET, PET&DX, and LOAD) with the baseline and GNN models.

Figure 5 shows that the AUC value obtained for the models is highly dependent on the split used. This fact is especially noticeable in the dataset with fewer patients, PET&DX. In this case, the baseline model is the one that obtains the widest distribution of all those shown, ranging from ~ 0.55 to more than 0.80. On the contrary, the GNN model obtains a much shorter and upwards shifted distribution. For PET and LOAD labelings, these boxplot distributions are similar in width although slightly shifted upwards in the case of GNNs. Regarding the average performance of each label, the difference between PET and LOAD is shallow (0.6801 ± 0.0643 and 0.6733 ± 0.0409, respectively), suggesting that these two labels are similar and more difficult to classify with the information provided than the PET&DX label (0.7521 ± 0.0589). Finally, as mentioned above, using only the APOE genotype as input in a logistic regression model led to results slightly better than GNNs, in all cases. Therefore, we must again emphasize the big influence of the APOE genotype in developing the three phenotypes evaluated in this work.

## Discussion

In this study, we present a novel pipeline for predicting the phenotype of a subject through the use of missense gene variants, included as node attributes in a biological network (Fig. 1a). Taking this into account, we built several graph datasets and labeled them with different phenotype labels as input for GNNs. The complete analysis presented in this work is based on the importance of using datasets organized in graphs and models that can operate on them to study complex and heterogeneous diseases^5^, such as neurodegenerative ones. Additionally, we performed several benchmarking steps (Fig. 1b) to corroborate the hypothesis that GNNs can be an advantage in phenotype prediction through genotype in Alzheimer’s Disease (AD).

We analyzed most of the results obtained in this manuscript concerning a baseline model that only included the APOE number of missense variants as input for Logistic Regression. Because AD is the most common NDD, its genetic architecture is well studied, with a remarkable breakthrough when the strong association of the APOE gene with the disease was discovered^33^. APOE is a major genetic risk for AD^19^, as several variants have been implicated in increased amyloid-beta deposition^34^. Therefore, given the importance and relationship of this gene with AD-related phenotypes, we decided to use it as a reference for more complex models such as GNNs.

As the first step in this work, we selected several biological networks from different sources to use as input for building their corresponding graph datasets. Next, we evaluated these graph datasets using GNNs for graph classification. We based the selection of the “best” biological network on comparing their corresponding GNN models’ performance against the previously mentioned baseline models (Fig. 2). This step was performed on the ADNI dataset, using two different labels named PET and PET&DX. In both label cases, the best-performing graph dataset came from the AD PPT-Ohmnet network (see Table 1). This PPI network involving genes related to AD is specific to brain tissue. It comes from a large tissue-specific PPI network where all the physical PPIs are supported by experimental evidence^35^. However, none of the GNN models with the different networks or labels obtained a significantly higher performance than their corresponding baseline models.

Within this comparison with the different biological networks and phenotypes considered, we noticed an increase in performance between PET and PET&DX classification greater in AD BioGRID than in the rest of the biological networks (Fig. 2). The explanation for this observed phenomenon is complex given that many agents play a role in classification: the genes and their variants, their interactions, and the specific topology of the network. Moreover, it is not easy to establish which set of genes and interactions are the ones that had the most weight in the phenotype prediction since the GraphGym^12^ framework does not allow exporting the generated GNN model to perform an analysis of the weights and a subsequent interpretability analysis. A possible explanation could be based on the possibility that there are nodes and edges in the AD BioGRID network that favor the classification of the PET-amyloid-positive dementia phenotype (PET&DX label, Fig. 2b). More specifically, these genes would add value in the sense of cognitive impairment (cases with dementia) apart from beta-amyloid accumulation. AD BioGRID has a total of nine genes (TFAM, PGRMC1, TNF, SLC30A6, SLC30A4, MPO, DHCR24, AMFR, and TPP1) that are not present in any of the other biological networks. However, as the differences in the patient graphs fall on the variants used, we performed a small search on the variants present in the genes mentioned in the ADNI dataset. Among these, we found rs1937 in the TFAM gene. This variant has previously specifically been associated with cognitive impairment (MMSE neurocognitive test) but not with beta-amyloid accumulation (CSF biomarkers)^36^. Evaluating the differences of these networks regarding different phenotypes is interesting for future work, although dependent on generating more interpretable models, as previously mentioned.

To verify if the small performance improvement of GNN w.r.t. the baseline model could be attributed to the information captured in the edges of the input network, we compared the results against other models that considered genotype information from the same gene set but did not use graph-structured data. For the prediction of the two considered labels (PET and PET&DX).

Firstly, none of the models performed significantly better than the baseline ones, although the GNN model in the PET&DX target was close to significance (p-value of 0.05172). There are several reasons why these GNN models did not obtain a better performance than the baseline model. First, there is a huge correlation between the rs429358 missense variant (present in the two cohorts used in this work) and the studied phenotypes. In fact, in a benchmarking study of machine learning models for predicting AD through genotyping the feature that all models selected as most important was this same missense variant present in APOE^37^. All other variants selected as important were either intronic or located in intergenic regions. Because we used PPI networks to structure the data in graph datasets in this work, we thought it convenient to use only coding missense variants in the first approach, as they are expected to have the strongest functional impact. However, the results obtained in this work and concerning the results reported in^37^, support the notion that additional risk factors of AD in addition to the APOE gene are likely found in non-coding variants, including those found in enhancer regions^38^. Another important reason may be that the representation of the genotype information in the network is too simplistic. Although this way of coding obtained good results in the prediction of Crohn's disease^9^, neural networks that directly encode sequence or structural information may be better suited.

Most non-GNN models were significantly lower in performance than the corresponding GNN models. When using the datasets with APOE, this was the case for all models except for Random Forest in the PET label. When using datasets without APOE, SVM RBF and Random Forest did not perform significantly lower than GNN in PET and PET&DX cases. These results indicate that the GNNs used in this work, while not yielding clinically relevant results in AD, have the potential for further exploration in disease prediction. When deleting the APOE gene from both the graph and tabular datasets employed, the performance of the models significantly dropped against models with APOE but did not obtain significantly worse values than random, except for the case of Logistic Regression in the PET&DX label. These obtained results highlight again the importance of missense variants in the APOE gene in the development of AD-related phenotypes.

We further sought to understand if the biological information coded in the edges of the network was used in prediction or if random networks with a similar topology could offer the same performance (Section “Using randomized networks as input”). In both label cases, PET and PET&DX, the original graph datasets obtained significantly better results than random graph datasets for both randomization methods (Supplementary Table 6). Therefore, interactions between genes and the overall topology of the specific brain PPI network played an important role in classification. Even though these results detected interactions and the original PPI network structure as an important factor in classification, one of the main limitations of this work is related to the framework used for the implementation of the GNNs. In the case of GraphGym, it is not possible to export the trained models, so we cannot perform a proper interpretability analysis.

Lastly, we obtained all these classification performances using different cohorts (ADNI and TGenII) for different classification labels. The results with GNNs with the different classification labels and input cohorts raise interesting questions about predicting different AD-related phenotypes through genotype information organized in a graph. In this work, we have considered three different targets: (i) amyloid PET status (PET), (ii) amyloid PET status together with diagnosis (PET&DX), and (iii) diagnosis (LOAD). From a neurological point of view, we can rank these phenotype labels according to their complexity or heterogeneity. The LOAD target is a classical case–control design in which only the diagnosis information is available. In the context of AD, this type of classification could include several misclassifications, such as pre-symptomatic AD cases as controls or other non-AD dementias diagnosed as AD. Due to this heterogeneity in clinical diagnosis labeling, it is also encouraged to use different endophenotypes related to the disease, such as amyloid-beta deposition. The use of endophenotypes improves phenotype definition and contributes to unraveling the etiology of AD^19^. This idea inspired us to use amyloid-PET status labeling in the ADNI dataset. However, the GNN performances (and also the baseline models) of PET and LOAD labels were very similar (0.67 and 0.68, respectively), even though their sample size was different (726 subjects for PET and 1599 for LOAD). Therefore we did not observe an improvement in phenotype prediction using AD diagnosis or amyloid PET status. In light of this, it is important to note that amyloid-beta deposition is not a specific biomarker for AD; therefore, subjects with different diagnoses and cognitive profiles could share the same amyloid-PET status. Accordingly, we created a subset of this target in the ADNI cohort, named PET&DX, using both amyloid-PET status and diagnosis. PET&DX target was the most homogeneous target as it only included patients diagnosed with AD with the positive amyloid-PET and subjects without cognitive decline with a negative amyloid-PET. These three targets’ characteristics are reflected in their classification results using GNNs. Consequently, classification is nearly significantly higher than the baseline model using a clearly-defined phenotype (PET&DX), genes previously associated with the disease (AD-related genes), and networks involving tissue-specific interactions (PPT-Ohmnet).

## Conclusions and outlook

Using AD-associated genes reported in DisGeNET, we have generated patient-specific graphs annotated with genotype information on missense variants. Different biological networks were considered to define edges in these networks which were then used in Graph Neural Networks (GNNs) for predicting phenotype labels. The results showed that GNNs did not highly outperform other non-GNN models, even a baseline model using the APOE genotype only. One of the main reasons for this could be that we ignored other types of variants as well as variants in non-coding regions of the DNA. However, in all cases, GNNs obtained the best results in terms of mean performance, demonstrating that the approach generally shows potential. Moreover, using random networks as input for GNN models confirmed that the interactions between the genes and overall topology graphs are important for the correct prediction. Therefore, organizing genotype data in a graph-structured dataset could significantly contribute to the phenotype prediction and understanding and unraveling of the network-level effect of the occurrence of several mutations in the development of disease. The results obtained depended on the biological network used as input for building the graph datasets and showed that it seems preferable to use networks related to the disease's affected tissue.

Because the results obtained with genetics and GNNs in predicting diseases as complex as AD are still disappointing, it appears necessary to define several lines of future work and possible topics for improvement in the proposed pipeline. (1) Firstly, as previously discussed, additional information needs to be coded in the graph as nodes or edges attributes. For example, stop-loss, stop gain, or splicing altering variants could be considered. PPI networks are limited in their most common form to the highest expressed form of the genes. Isoforms can have a significant impact on the PPI and thus on this pipeline^39^. (2) Secondly, another reason could be the lack of feature selection in the presented methodology. Including pathogenicity criteria could give more or less weight to the different variants, therefore, providing more information on how this network is perturbed and orienting the model towards the specific phenotype to be predicted. (3) Thirdly, the way that we coded the variants as node attributes does not include other important genetic phenomena such as epistasis or epigenetic mechanisms of gene regulation. Both mechanisms likely play an important role in the genomics of complex phenotypic traits^38,40,41^. (4) Finally, one of the most challenging tasks is to develop a more interpretable neural network model. For this, tools such as GraphGym give the scientific community the ability to test and evaluate complex models such as GNNs in a user-friendly environment. However, it is also important to develop *ad-hoc* models to extract how these complex neural network models work on the dataset.

Finally, it is interesting to note that this work is not at odds with further exploration of results. The GNN models could use larger biological network-based graph datasets that include neighbors of the selected genes (nodes) of interest. However, it is crucial to take into account the sample size. If many genes per individual are included, a dimensionality problem could arise from which it is not easy to extract information without overfitting. In addition, it must be considered that the higher the dimensionality, the higher the computational cost of the models' training. Therefore, we decided to conduct this first work in a reduced search space, using genes previously related to Alzheimer's disease. The ideal scenario would be where we find as few genes or variants as possible organized in a network to predict the phenotype accurately.

In conclusion, we have carried out an exhaustive benchmarking work on using the very novel and actual GNNs to predict phenotype through genotype in the context of a highly complex disease such as AD. While in our study GNNs could not deliver clinically relevant results for the early diagnosis of AD, we consider it valuable to take these first steps towards phenotype prediction by structuring genomic data as graphs.

## Supplementary Information

Supplementary Tables.

## Data Availability

The Alzheimer’s Disease Neuroimaging Initiative (ADNI) data underlying this article are available at the ADNI website upon formal request: adni.loni.usc.edu. The TGen II data underlying this article are available at NIAGADS upon formal request: https://www.niagads.org/datasets/ng00028.
