# Supplementary material for: On the limits of graph neural networks for the early diagnosis of Alzheimer’s disease
Source: Sci Rep. 2022 Oct 21;12:17632. doi: 10.1038/s41598-022-21491-y (PMC9587223; doi:10.1038/s41598-022-21491-y)
Supplement: Supplementary file 1 — Supplementary Tables. [file 41598_2022_21491_MOESM1_ESM.pdf]

On the limits of graph neural networks for the early diagnosis of Alzheimer's Disease

Laura Hernández-Lorenzo[1,2,3,*], Markus Hoffmann[3,4], Evelyn Scheibling[3], Markus List[3, †], Jordi A. Matías-Guiu[2, †], Jose L. Ayala[1, †], and for the Alzheimer's Disease Neuroimaging Initiative[**]

[1]Department of Computer Architecture and Automation, Computer Science Faculty, Complutense University of Madrid, 28040 Madrid, Spain
[2]Department of Neurology, Hospital Clínico San Carlos, San Carlos Research Health Institute (IdISSC), Universidad Complutense, 28040 Madrid, Spain
[3]Big Data in BioMedicine Group, Chair of Experimental Bioinformatics, TUM School of Life Sciences, Technical University of Munich, Munich, Germany
[4]Institute for Advanced Study (Lichtenbergstrasse 2 a, D-85748 Garching, Germany), Technical University of Munich, Germany;

** corresponding author: laurahl@ucm.es*

† The authors wish it to be known that, in their opinion, the last three authors should be regarded as Joint Senior Authors.
*** Data used in the preparation of this article were obtained from the Alzheimer's Disease Neuroimaging Initiative (ADNI) database (adni.loni.usc.edu). As such, the investigators within the ADNI contributed to the design and implementation of ADNI and/or provided data but did not participate in the analysis or writing of this report. A complete listing of ADNI investigators can be found at:*
https://adni.loni.usc.edu/wp-content/uploads/how_to_apply/ADNI_Acknowledgement_List.pdf

Supplementary Information

1. Materials and Methods

1.1. GraphGym description and use

As is the case in many deep learning tasks, one of the main problems is searching for the best hyperparameters and network architecture. Because the datasets built in this work were new, we employed the novel tool called GraphGym developed by [12] for searching for the best GNN architecture for each classification task. Using configuration and grid files, it is possible to launch a batch of experiments in a relatively short search time. Applying one GNN design or model to a specific task is defined as an experiment in this framework. Several design dimensions (hyperparameters that can be tuned, e.g., batch normalization) with different options (e.g., True) conform to the so-called design space. Three designs can be distinguished: intra-layer, inter-layer, and learning configuration, where "layer" refers to message passing layers. Intra-layer design corresponds to dimensions that could vary within the message passing layers. In this case, the only dimension that could vary was the aggregation function (Mean, Max, or Sum). Inter-layer design contains the dimensions that could change between the message passing layers. These dimensions include the number of message passing layers, the type of skip connection that exists between them (Skip-Sum or Skip-Concat), or the number of layers that could have the multilayer perceptrons (MLPs) before and after the message passing layers (pre-process and post-process MLP, respectively). Finally, the learning configuration design contains four basic dimensions for any neural network training: batch size, learning rate, optimizer, and the maximum number of training epochs. All dimensions here described, except

for the number of epochs and message passing layers, were set to the values or options found to be preferable for an ample task space using GNNs by [12]. Supplementary Table 1 shows the design space used for each classification task performed in this work.

Supplementary Table 1. Design space for each classification task in GraphGym

Design	Dimension	Options
Intra-layer	Batch normalization<br>Dropout<br>Activation function<br>Aggregation function	True<br>False<br>PReLU<br>Mean, Max, Sum
Inter-layer	Pre-process MLP layers<br>Layer-connectivity Message-passing layers<br>Post-process MLP layers	1, 2<br>Skip-Sum, Skip-Concat<br>**2**<br>2, 3
Learning configuration	Batch size<br>Learning rate<br>Optimizer<br>Training epochs	32<br>0.01<br>Adam<br>**200**

Bolded values are configuration parameters changed from original work by You et al. (2020).

Supplementary Table 2. Grid of hyperparameters used for each canonical machine learning algorithm.

Algorithm	Hyperparameters	Grid values
Logistic Regression	NA	NA
SVM Linear	kernel<br>C	'linear'<br>0.01, 0.1, 1, 10, 100, 1000
SVM RBF	kernel<br>C<br>gamma	'rbf'<br>0.01, 0.1, 1, 10, 100, 1000<br>0.001, 0.01, 0.1, 1
Random Forest	n_estimators	50, 500, 5000

Note: All other values were left as default according to Scikit-Learn v.1.0.2 library[31].

2. Results

Supplementary Table 3. GNN best configurations and their classification metrics obtained in ADNI dataset test set using different biological networks as input.

(a) PET label results

Network	Best GNN configuration	Accuracy	Precision	Recall	F1-Score	AUC-ROC
AD BioGRID	2, 2, 2, Skip-Cat, Max	0.6275 ± 0.0511	0.662 ± 0.0402	0.7260 ± 0.1274	0.6812 ± 0.0654	0.6648 ± 0.0656
AD GIANT	1, 2, 3, Skip-Sum, Sum	0.6078 ± 0.0713	0.6823 ± 0.0796	0.6716 ± 0.1828	0.6340 ± 0.1102	0.6635 ± 0.0786
AD HuRI	2, 2, 2, Skip-Concat, Sum	0.6367 ± 0.0652	0.7019 ± 0.0614	0.6439 ± 0.1086	0.6628 ± 0.0693	0.6692 ± 0.0624
AD PPT-Ohmnet	2, 2, 2, Skip-Sum, Max	0.6362 ± 0.0748	0.6739 ± 0.1139	0.6488 ± 0.1169	0.6540 ± 0.1031	0.6801 ± 0.0643
AD STRING	1, 2, 2, Skip-Concat, Sum	0.6407 ± 0.0576	0.6642 ± 0.0492	0.7675 ± 0.0823	0.7035 ± 0.0507	0.6763 ± 0.0637

(b) PET&DX label results

Network	Best GNN configuration	Accuracy	Precision	Recall	F1-Score	AUC-ROC
AD BioGRID	1, 2, 2, Skip-Concat, Mean	0.6827 ± 0.1097	0.6991 ± 0.1272	0.7249 ± 0.2027	0.6915 ± 0.1773	0.7526 ± 0.0819
AD GIANT	2, 2, 2, Skip-Concat, Sum	0.6442 ± 0.0705	0.7022 ± 0.1116	0.6588 ± 0.1781	0.6561 ± 0.1334	0.7035 ± 0.0634
AD HuRI	1, 2, 3, Skip-Sum, Sum	0.6824 ± 0.0628	0.7228 ± 0.0641	0.7502 ± 0.103	0.7291 ± 0.0553	0.7397 ± 0.0682
AD PPT-Ohmnet	2, 2, 2, Skip-Concat, Max	0.6992 ± 0.0683	0.7381 ± 0.0688	0.7552 ± 0.1105	0.7408 ± 0.0611	0.7521 ± 0.0589
AD STRING	1, 2, 2, Skip-Sum, Max	0.6678 ± 0.0448	0.7036 ± 0.0774	0.7894 ± 0.1497	0.7266 ± 0.0469	0.7502 ± 0.0563

GNN configuration is presented as pre-MLP layers, message-passing layers, post-MLP layers, layer connectivity, and aggregation function. All performance values are presented as the mean of the classification metric ± standard deviation.

Supplementary Table 4. GNN and non-GNN models classification metrics obtained in the ADNI cohort test set using different datasets as input: only using APOE, several genes in AD PPT-Ohmnet network, and those same genes without including APOE gene.

(a) PET label results

Dataset	Model	Accuracy	Precision	Recall	F1-Score	AUC
Only APOE	Baseline model	0.5992 ± 0.0399	0.6345 ± 0.0686	0.7659 ± 0.2127	0.6725 ± 0.0719	0.6406 ± 0.0691
AD PPT-Ohmnet	Logistic Regression	0.599 ± 0.0534	0.6331 ± 0.0515	0.722 ± 0.156	0.6644 ± 0.066	0.6229 ± 0.0693
	SVM Linear	0.6074 ± 0.0553	0.6549 ± 0.0646	0.7 ± 0.1879	0.6604 ± 0.0688	0.627 ± 0.0493
	SVM RBF	0.6006 ± 0.061	0.6206 ± 0.079	0.8683 ± 0.1858	0.7062 ± 0.0506	0.6275 ± 0.0628
	Random Forest	0.6197 ± 0.0622	0.6799 ± 0.0612	0.6244 ± 0.1017	0.647 ± 0.0673	0.6291 ± 0.0771
	GNN GraphGym	0.6362 ± 0.0748	0.6739 ± 0.1139	0.6488 ± 0.1169	0.654 ± 0.1031	**0.6801 ± 0.0643**
AD PPT-Ohmnet no APOE	Logistic Regression	0.5538 ± 0.0176	0.5598 ± 0.0095	0.9805 ± 0.0277	0.7127 ± 0.0147	0.481 ± 0.067
	SVM Linear	0.5607 ± 0.016	0.5631 ± 0.0087	0.9902 ± 0.0236	0.7179 ± 0.0129	0.4942 ± 0.0459
	SVM RBF	0.5593 ± 0.0151	0.5625 ± 0.0086	0.9878 ± 0.0237	0.7168 ± 0.0124	0.5192 ± 0.0726
	Random Forest	0.5524 ± 0.027	0.5602 ± 0.0144	0.9634 ± 0.035	0.7084 ± 0.0202	0.4853 ± 0.0771
	GNN GraphGym	0.5372 ± 0.0321	0.5314 ± 0.1073	0.7821 ± 0.2263	0.586 ± 0.1563	**0.5454 ± 0.0513**

(b) PET&DX label results

Dataset	Model	Accuracy	Precision	Recall	F1-Score	AUC
Only APOE	Baseline model	0.7066 ± 0.0682	0.7556 ± 0.0714	0.7368 ± 0.1018	0.7412 ± 0.0615	0.6901 ± 0.0979
AD PPT-Ohmnet	Logistic Regression	0.6627 ± 0.0798	0.6977 ± 0.0777	0.7424 ± 0.109	0.715 ± 0.0707	0.6825 ± 0.076
	SVM Linear	0.6531 ± 0.0541	0.6987 ± 0.0651	0.7319 ± 0.1307	0.7053 ± 0.0463	0.6872 ± 0.0654
	SVM RBF	0.669 ± 0.0637	0.7172 ± 0.0779	0.7371 ± 0.1248	0.7174 ± 0.0491	0.7025 ± 0.0638
	Random Forest	0.6906 ± 0.0579	0.7414 ± 0.0473	0.7149 ± 0.1135	0.7231 ± 0.0638	0.6901 ± 0.075
	GNN GraphGym	0.6992 ± 0.0683	0.7381 ± 0.0688	0.7552 ± 0.1105	0.7408 ± 0.0611	**0.7521 ± 0.0589**
AD PPT-Ohmnet no APOE	Logistic Regression	0.568 ± 0.0294	0.5737 ± 0.0192	0.9617 ± 0.0455	0.7184 ± 0.0247	0.4425 ± 0.0725
	SVM Linear	0.5586 ± 0.0204	0.5701 ± 0.0158	0.9398 ± 0.0607	0.7090 ± 0.0224	0.4687 ± 0.0918
	SVM RBF	0.5711 ± 0.0188	0.5758 ± 0.0145	0.9617 ± 0.0587	0.7197 ± 0.0205	**0.5571 ± 0.0481**
	Random Forest	0.5679 ± 0.0307	0.5760 ± 0.0190	0.9339 ± 0.0684	0.7119 ± 0.0309	0.5122 ± 0.0916
	GNN GraphGym	0.5502 ± 0.033	0.5741 ± 0.0413	0.8139 ± 0.1811	0.6416 ± 0.0979	0.5554 ± 0.0485

Supplementary Table 5. 1-sample t-test p-values obtained comparing each model's performance against the baseline model performance (Logistic Regression with only APOE as input) and against a random value performance (AUC 0.5).

Label	Dataset	Model	Against baseline	Against GNN	Against random	Against no APOE
PET	AD PPT-Ohmnet	Logistic Regression	7.1252e-01	**3.5778e-02 ***	9.9983e-01	**9.8484e-05 ***
		SVM Linear	6.8956e-01	**2.6525e-02 ***	9.9999e-01	**3.5104e-06 ***
		SVM RBF	6.6811e-01	**4.0317e-02 ***	9.9994e-01	**1.0920e-03 ***
		Random Forest	6.3489e-01	6.2651e-02	9.9975e-01	**2.8652e-04 ***
		GNN GraphGym	1.0072e-01	-	1.0000e+00	**3.1765e-05 ***
	AD PPT-Ohmnet no APOE	Logistic Regression	9.9997e-01	**1.3357e-02 ***	1.9665e-01	-
		SVM Linear	9.9999e-01	**1.5148e-02 ***	3.4927e-01	-
		SVM RBF	9.9939e-01	1.8130e-01	7.8729e-01	-
		Random Forest	9.9992e-01	**2.7457e-02 ***	2.8052e-01	-
		GNN GraphGym	9.9871e-01	-	9.8960e-01	-
PET&DX	AD PPT-Ohmnet	Logistic Regression	5.7567e-01	**1.7280e-02 ***	9.9998e-01	**5.0849e-07 ***
		SVM Linear	5.2998e-01	**1.5860e-02 ***	1.0000e+00	**4.2853e-06 ***
		SVM RBF	3.7087e-01	**4.3754e-02 ***	1.0000e+00	**9.3743e-06 ***
		Random Forest	4.9994e-01	**2.7398e-02 ***	9.9999e-01	**7.9737e-05 ***
		GNN GraphGym	5.1717e-02	-	1.0000e+00	**9.4311e-08 ***
	AD PPT-Ohmnet no APOE	Logistic Regression	1.0000e+00	**3.4273e-04 ***	**1.6727e-02 ***	-
		SVM Linear	9.9997e-01	**8.2945e-03 ***	1.5417e-01	-
		SVM RBF	9.9942e-01	5.3144e-01	9.9773e-01	-
		Random Forest	9.9973e-01	1.0200e-01	6.5781e-01	-
		GNN GraphGym	9.9947e-01	-	9.9717e-01	-

Against baseline, H1: mean other model is greater than mean baseline
Against GNN, H1: mean other non-GNN model is lower than mean GNN model
Against random, H1: mean model is greater than a random AUC value
Against no APOE, H1 mean no APOE model is lower than mean APOE model
*p-values < 0.05

Supplementary Table 6. 1-sample t-test p-values adjusted by Benjamini-Hochberg method [33] obtained by comparing original graph datasets' performance *vs.* random graph datasets' performance on the test set using PET and PET&DX labels on each of their corresponding folds.

Label - Fold number	p-value “Shuffled”	p-value “Rewired”
PET - Fold 1	2.6017e-23 *	3.7085e-24 *
PET - Fold 2	4.5905e-10 *	1.4036e-12 *
PET - Fold 3	4.5723e-32 *	7.3250e-33 *
PET - Fold 4	5.1310e-09 *	4.8923e-10 *
PET - Fold 5	1.6292e-16 *	2.0698e-18 *
PET - Fold 6	3.7464e-03 *	1.3709e-04 *
PET - Fold 7	1.5944e-26 *	2.1551e-25 *
PET - Fold 8	9.6640e-01	9.8883e-01
PET - Fold 9	1.6147e-01	6.9903e-02
PET - Fold 10	1.6726e-21 *	1.6305e-22 *
PET&DX - Fold 1	2.1960e-33 *	1.9714e-34 *
PET&DX - Fold 2	2.6590e-54 *	3.0006e-57 *
PET&DX - Fold 3	1.3214e-07 *	5.5099e-06 *
PET&DX - Fold 4	6.1280e-37 *	2.0128e-36 *
PET&DX - Fold 5	1.5999e-23 *	1.0105e-22 *
PET&DX - Fold 6	5.5533e-11 *	9.8836e-12 *
PET&DX - Fold 7	8.9045e-06 *	3.9243e-02 *
PET&DX - Fold 8	1.0000e+00	1.0000e+00
PET&DX - Fold 9	4.1115e-43 *	2.2059e-41 *
PET&DX - Fold 10	9.0603e-31 *	1.2288e-29 *

*p-values < 0.05.

Supplementary Table 7. GNN best configuration and their performance results obtained in LOAD dataset test set using graph datasets obtained with AD PPT-Ohmnet PPI network.

Network	Best GNN configuration	Accuracy	Precision	Recall	F1-Score	AUC-ROC
AD PPT-Ohmnet	2, 2, 3, Skip-Concat, Sum	0.6523 ± 0.0486	0.6917 ± 0.1004	0.7781 ± 0.0928	0.7236 ± 0.0797	**0.6733 ± 0.0409**

GNN configuration is presented as pre-MLP layers, message-passing layers, post-MLP layers, layer connectivity, and aggregation function. All performance values are presented as the mean of the classification metric ± standard deviation.

Supplementary Table 8. GNN and non-GNN models classification metrics obtained in the LOAD cohort test set using different datasets as input: only using APOE, several genes in AD PPT-Ohmnet network, and those same genes without including APOE gene.

Dataset	Model	Accuracy	Precision	Recall	F1-Score	AUC
Only APOE	Baseline model	0.6648 ± 0.0397	0.7662 ± 0.0341	0.6784 ± 0.0466	0.7191 ± 0.0373	0.6591 ± 0.0451
AD PPT-Ohmnet	Logistic Regression	0.6542 ± 0.0335	0.7353 ± 0.0261	0.7109 ± 0.0491	0.7222 ± 0.032	0.6541 ± 0.0505
	SVM Linear	0.6535 ± 0.04	0.6962 ± 0.035	0.8135 ± 0.0915	0.7472 ± 0.0373	0.6482 ± 0.0465
	SVM RBF	0.6485 ± 0.028	0.6747 ± 0.066	0.9131 ± 0.1413	0.7649 ± 0.0243	0.659 ± 0.0564
	Random Forest	0.6604 ± 0.0405	0.713 ± 0.0267	0.778 ± 0.0623	0.7431 ± 0.0373	0.6539 ± 0.0464
	GNN GraphGym	0.6523 ± 0.0486	0.6917 ± 0.1004	0.7781 ± 0.0928	0.7236 ± 0.0797	**0.6733 ± 0.0409**

Supplementary Table 9. 1-sample t-test p-values obtained comparing each model's performance against the baseline model performance (Logistic Regression with only APOE as input) and against a random value performance (AUC 0.5).

Label	Dataset	Model	Against baseline	Against random
LOAD	AD PPT-Ohmnet	Logistic Regression	5.9103e-01	**7.6678e-09 ***
		SVM Linear	6.9812e-01	**3.8991e-09 ***
		SVM RBF	5.0101e-01	**2.5141e-08 ***
		Random Forest	5.9751e-01	**2.0959e-09 ***
		GNN GraphGym	2.3507e-01	**4.2666e-11 ***

*p-values < 0.05
